# Self-Reflected Well-Being via a Smartphone App in Clinical Medical Students: Feasibility Study

**DOI:** 10.2196/mededu.9128

**Published:** 2018-03-07

**Authors:** Elizabeth K Berryman, Daniel J Leonard, Andrew R Gray, Ralph Pinnock, Barry Taylor

**Affiliations:** ^1^ Dunedin School of Medicine University of Otago Dunedin New Zealand

**Keywords:** mental health, medical students, medical education, bullying, teaching, mhealth

## Abstract

**Background:**

Well-being in medical students has become an area of concern, with a number of studies reporting high rates of clinical depression, anxiety, burnout, and suicidal ideation in this population.

**Objective:**

The aim of this study was to increase awareness of well-being in medical students by using a smartphone app. The primary objective of this study was to determine the validity and feasibility of the Particip8 app for student self-reflected well-being data collection.

**Methods:**

Undergraduate medical students of the Dunedin School of Medicine were recruited into the study. They were asked to self-reflect daily on their well-being and to note what experiences they had encountered during that day. Qualitative data were also collected both before and after the study in the form of focus groups and “free-text” email surveys. All participants consented for the data collected to be anonymously reported to the medical faculty.

**Results:**

A total of 29 participants (69%, 20/29 female; 31%, 9/29 male; aged 21-30 years) were enrolled, with overall median compliance of 71% at the study day level. The self-reflected well-being scores were associated with both positive and negative experiences described by the participants, with most negative experiences associated with around 20% lower well-being scores for that day; the largest effect being “receiving feedback that was not constructive or helpful,” and the most positive experiences associated with around 20% higher scores for that day.

**Conclusions:**

The study of daily data collection via the Particip8 app was found to be feasible, and the self-reflected well-being scores showed validity against participant’s reflections of experiences during that day.

## Introduction

### Background

There is an increasing number of studies that have suggested that medical students experience high rates of depression and suicidal ideation [1]. A systematic review conducted in 2016 by Rotenstein et al from 167 cross-sectional studies (n=116,628) and 16 longitudinal studies (n=5728) from 43 countries found that depressive symptom prevalence is substantially higher among medical students than among individuals of similar age in the general population. The finding in the longitudinal analysis of this review showed an increase in depressive symptom prevalence with the onset of medical school. The overall pooled crude prevalence of depression or depressive symptoms was 27.2%, compared with 2 large representative epidemiological studies, which estimated depressive symptom prevalence in nonmedical students ranging from 13.8% to 21.0% [1,2].

Furthermore, the Australian National Mental Health Survey of Doctors and Medical Students showed that approximately one in 5 medical students (20%) had thoughts of suicide in the previous 12 months [3]. Similarly, the Rotenstein review showed a prevalence rate for suicidal ideation, extracted from 24 cross-sectional studies (n=21,002) from 15 countries, of one in 10 medical students. Currently, there are no available data on suicide rates in medical students. However, two systematic reviews of qualified doctor suicides conducted by Schernhammer and Colditz in 2004 and Damasceno et al in 2017 revealed the aggregate suicide rate ratio for male doctors, compared with the general population, was 1.41. For female doctors in the same studies, the ratio was 2.27 [4].

Many factors contribute to poor well-being and may include occupational factors, emotionally demanding situations, unrealistic expectations, and confrontations with illness, death, and dying [5-8]. Degrading experiences such as bullying or harassment at work have been shown to be associated with suicidal thoughts [9]. The New Zealand Medical Students’ Association (NZMSA) surveyed their members in 2015 and reported that 54% had experienced bullying or sexual harassment while on clinical placement [10]. It has been suggested that sometimes accusations of bullying can be linked to situations that are an inevitable part of training [7]. For example, trainers giving feedback to trainees that they are not performing at the expected level [11]. However, research has clearly shown that perceived mistreatment regardless of the intention of the perpetrator is viewed by medical students as a major source of stress and well-being depletion [5,12].

Due to the reported high prevalence of depressive and suicidal thoughts in medical students, there is a need for additional research to identify the root causes of emotional distress. Recommendations from past studies have suggested adopting prospective study designs, so that the same individuals can be assessed over time [1].

### Objectives

The primary objective of this study was to assess the feasibility of utilizing a smartphone app, such as the Particip8 app, for the collection of students’ individual self-reflected experiences and sense of individual well-being. Secondary objectives were to correlate daily experiences with the self-reflected well-being score and to assess the use of the “safety pop-up feature” in prompting students to access help at an earlier stage. Qualitative data were also collected to assess the effectiveness of the Particip8 app in increasing self-awareness of well-being.

## Methods

### Study Design

**Figure 1:**
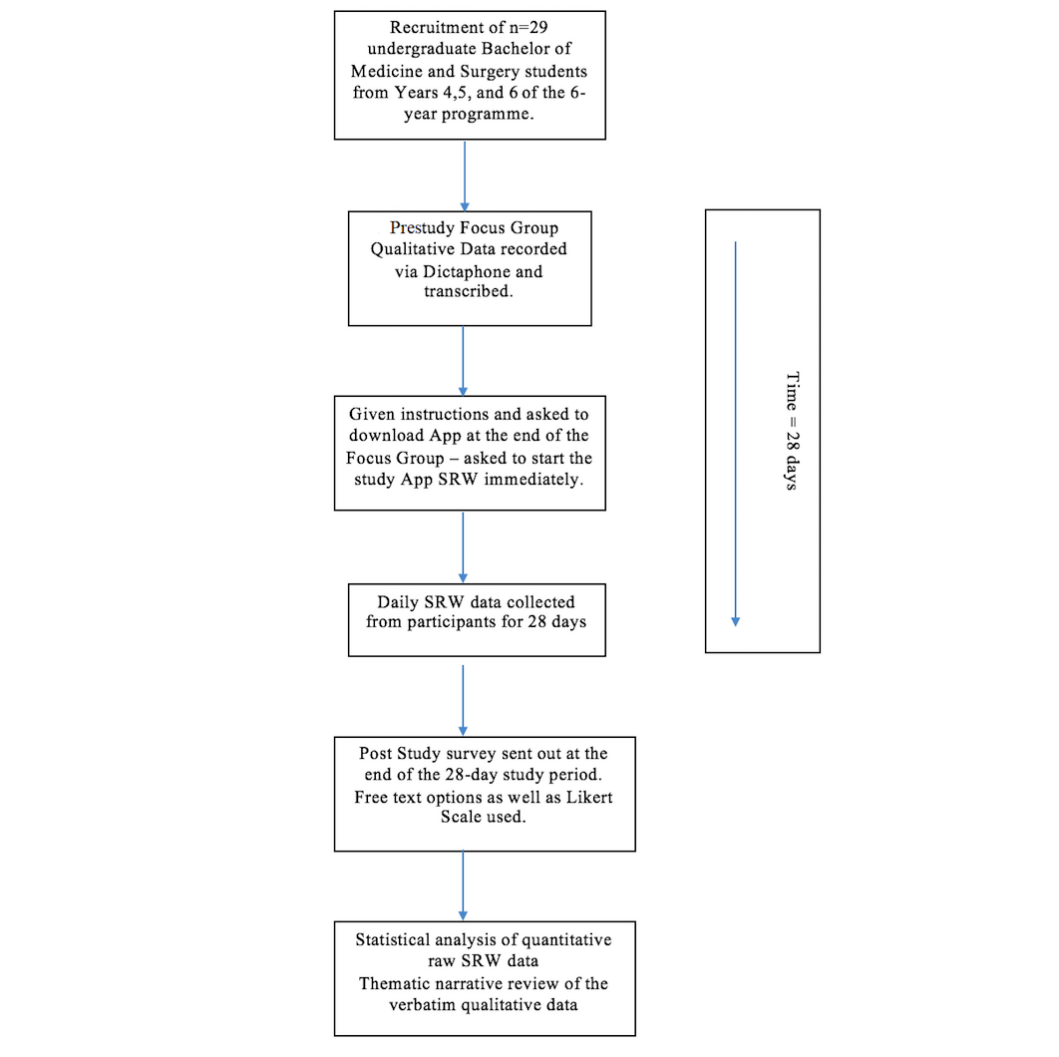
Methodology flowchart. SRW: self-reflected well-being.

The methodology used for this feasibility study was a mixed qualitative and quantitative approach. The quantitative aspect utilizes the ecological momentary assessment (EMA) methods as described by Shiffman et al [13]. The qualitative data were based on grounded theory methodology and analyzed with a narrative thematic approach based on descriptions in Glaser and Strauss (2017) and Braun and Clarke (2006), respectively [14,15]. The data for the qualitative analysis were obtained from prestudy focus groups, as well as poststudy email surveys. An overview of the methodology is set out in Figure 1. The necessary sample size for the feasibility component of the study was determined to be 30 participants, and the duration of the study was determined to be 28 days. This was to allow sufficient opportunities for participants to explore the Particip8 app under different conditions and to achieve effective saturation of their experiences. Participants were asked to use the Particip8 app on a daily basis to record their self-reflection on well-being. Participants were able to select a face emoticon scale to indicate how they felt on that particular day. Additionally, participants were also asked to select from a list provided, the experiences that they had been exposed to during that day. Participants could choose multiple experiences for the day; however, they could only log one self-reflected well-being score.

Consultation with key stake holders was undertaken before applying for ethics approval. These key stake holders included the Pro-Vice Chancellor of Health Sciences, the Dean of Māori, and the Dean of Pacifica. Other key stakeholders such as student groups that included the Otago University Medical Students Association, Te Oranga Aotearoa, NZMSA, and the Pacific Island Health Professional Student Association were also consulted. Feedback from these stakeholders was taken into consideration during the study, and as a result, previous aspects were changed and amended. Ethics approval was granted by the University of Otago Head of Department (Ethics no. D16/308).

### Participant Recruitment

The participants were students recruited from the Dunedin School of Medicine. Recruitment was conducted via posters in student areas, lecture announcements, and through social media posts. A total of 29 students voluntarily applied for the study, and all 29 students met the inclusion criteria. As a result, all 29 students were enrolled in the study. The inclusion criteria included (1) The ownership of a personal Android or apple smartphone device; (2) Enrolled at the Dunedin School of Medicine in the bachelor of medicine and surgery (MBChB) degree; (3) Were currently in their 4th, 5th, or 6th year of the undergraduate MBChB program; and (4) Currently undertaking a clinical placement. The participants were provided with a code for downloading the Particip8 app once they had inquired about the study, or when they attended the prestudy focus group. A webcast was made available to students with instructions on how to download and use the Particip8 app. Both iPhone operating system (iOS, Apple Inc) and Android platforms were made available. The Particp8 app was not available for those who owned a Windows phone, or for nonsmartphone mobile devices.

The app was named “Particip8” because students were required to be active participants in their own well-being. There were eight daily questions that the students were required to answer. For example, one question asked what placement the student was on that day, another question had five questions from the World Health Organization’s (five) Well-Being Index (WHO-5), and other questions related to the experiences that the student had experienced that day.

Upon downloading the Particip8 app, the initial log-in page had the details of the study and asked for the participant’s consent to participate in the study. All participants gave consent via the Particip8 app to be in the study. Participants were offered no financial incentives or reimbursement for participation in the study. The second page of the Particip8 app required each participant to enter their baseline demographic data such as their age, entry into medical school (either from secondary school after completing the health science first year (HSFY) program, after completing a previous degree (postgraduate), or from the “other” category that included those who have an undergraduate or postgraduate degree and have worked in allied health for a minimum of 5 years, ethnicity, as well as their gender. The webcast that had the download instructions also had instructions on how to complete the survey and how to customize the time setting for the daily push notification reminder.

### Evaluation of the Screening Tool Used

The Particip8 app was specifically developed for clinical medical students, by clinical medical students. It involved using an international validated survey, the WHO-5. The question wording and order in the WHO-5 did not change. The time period of interest, however, was changed from “the last 14 days,” to asking “the last 24 hours” to suit daily recording. This adaptation was reviewed by the New Zealand World Health Organization Quality of Life Group and deemed suitable to be used in this shortened time frame. Notably, however, no other published research has used it as a daily survey before. The WHO-5 was chosen because it has a sensitivity of 0.93 and a specificity of 0.83 in the detection of depression [14].

**Figure 2:**
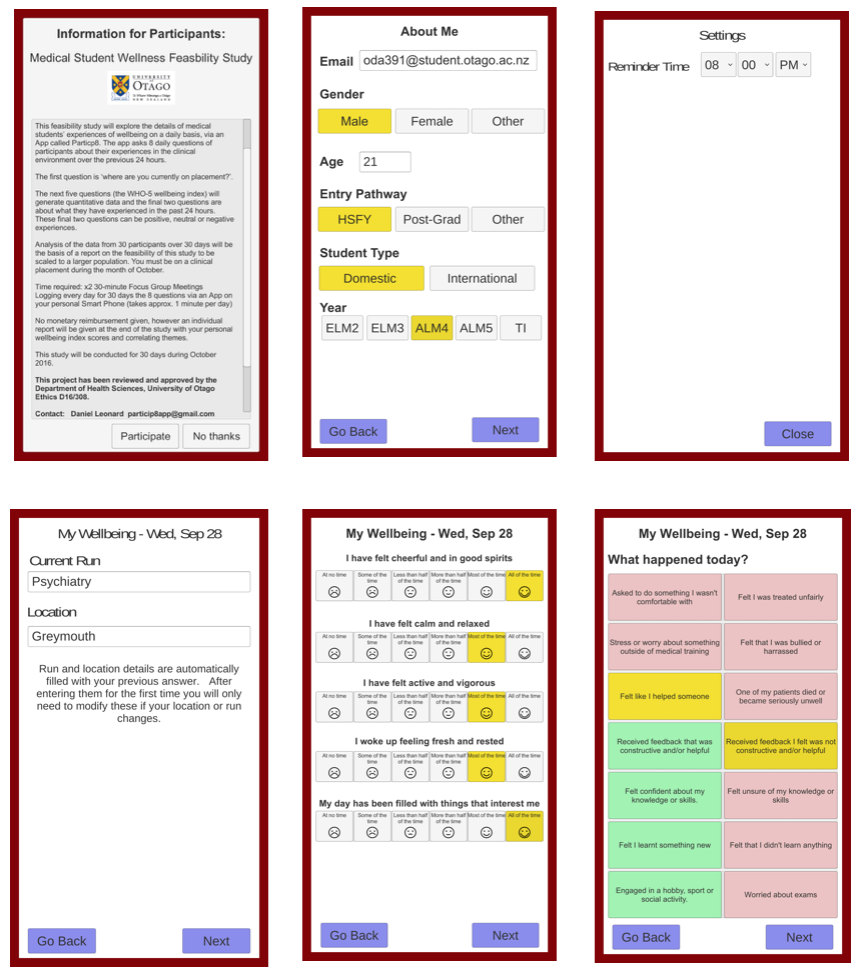
Screenshots of the app.

 The Particip8 app asked the WHO-5 questions daily and utilized the visual aid of a facial emoticon scale. The emoticon scale was selected to help with ease, speed, and accuracy of answering the five questions. The next page of the Particip8 app was a list of experiences that the student possibly could have experienced during the day. These experiences were chosen from the “NZMSA 2015 Bullying and Harassment Survey” and commonly experienced situations of clinical medical students [10]. Screenshots from the Partcip8 app are presented in Figure 2.

### Statistical Methods

The size of the study, being a total of 30 participants, was determined to be sufficient for the quantitative component. The size of the study would provide sufficiently precise estimates for standard deviations, correlations between repeated measures, and rates and patterns of missing data for designing larger studies in the future.

Appropriate summary statistics were calculated for all variables of interest. Analysis included linear mixed models (LMMs), with a random participant effect to accommodate the repeated measures over the study days. The LMMs were used to examine associations between each of the WHO-5 items and its combined score and each of the situational (day of week, location), experiential (eg, learning something new), demographic (age, gender, ethnicity, and entry pathway), and study-related (day of study and delay before reporting) variables. Model diagnostics included examining model residual normality and homoscedasticity. Subsequently, the experiential variables (yes or no) were examined for associations with the situational, demographic, and study-related variables by using mixed logistic regression models. All statistical analyses were conducted using Stata (StataCorp) 14.2, with two-sided *P*<.05 considered statistically significant in all cases. No formal adjustment was made for the multiple comparisons, and marginal results should be interpreted with caution.

## Results

### Participant Characteristics

A total of 29 participants were analyzed in this study. Participant characteristics (Table 1) showed the age range from 21 to 29 years, with a median age of 23 years (starting at age 19 years with high school leavers completing HSFY entry method, the majority of students would be either aged 21 years or above in 4th year medical school). The majority of participants (69%, 20/29) identified as female, with the remaining participants (31%, 9/29) identifying as male. Ethnicity was 24% (7/29) Māori, 38% (11/29) New Zealand European, and 38% (11/29) identifying as “other” (Indian, Sri Lankan, Chinese, South East Asian, and Pacific Islander). Entry pathway and student type and gender percentage were both reflective of the cohort group from which the participants were selected.

**Table 1:** Participant demographics.

Variable			n (%)
**Student-level**			
	**Age (years)**		
		21	3 (10)
		22	6 (21)
		23	8 (28)
		24	4 (14)
		25	3 (10)
		25+	5 (17)
	**Gender**		
		Female	20 (69)
		Male	9 (31)
	**Year**		
		Advanced learning in medicine (ALM) 4th year	15 (52)
		ALM 5th year	5 (17)
		Trainee intern	9 (31)
	**Entry pathway**		
		Health science first year program	21 (72)
		Other	2 (7)
		Postgraduate	6 (21)
	**Student type**		
		Domestic	26 (90)
		International	3 (10)
	**Prioritized ethnicity**		
		Māori	7 (24)
		European	11 (38)
		Other	11 (38)

### App Feasibility Analysis

Compliance was measured as completing the daily survey within the allotted time period. In total, 471 days were completed over the 28-day study period, resulting in an overall median compliance rate of 71% at the day level. A total of 13 participants (45%, 13/29) completed 80% to 100% of the days, achieving the required compliance threshold for EMA studies; 4 (14%, 4/29) completed between 50% to 80% of days; and 12 participants (41%, 12/29) completed less than 50% of the days. The longitudinal data showed that compliance rates steadily declined over the 28 days (data not shown. Females were nonstatistically significantly more compliant than men, and there was no evidence for associations with compliance for any of the other student demographics (age group, ethnicity, or year of study).

### Well-Being by Day of the Week and Type of Day

For the overall score (Multimedia Appendix 1), there were differences between various days of the week (overall *P*=.003). Tuesday had the lowest overall well-being mean (2.91/.00), and Saturday had the highest mean (3.51/5.00). The same pattern was observed for the five individual questions, but only feeling cheerful (Q1), waking up fresh and rested (Q4), and day filled with things that interested (Q5) were statistically significantly different by the particular day of the week. Days off (mean=3.44) had higher well-being scores than days where the students attended placements (3.01, *P*<.001). Four of the questions showed statistically significant differences, except feeling active and vigorous (Q3).

### Ethnicity

Māori students were 1.22 times more likely to engage in sports, social activities, or hobbies than New Zealand European students and 2.27 times more likely compared with non-New Zealand European students (overall test for ethnicity *P*=.009, results not shown).

### Entry Pathway Into Medicine

Postgraduate and “other” students were 2.2 times more likely to feel that they “did not learn anything” (19.5% vs 8.9%, *P*=.02). Postgraduate and “other” students were 2.5 times more likely to feel more unsure of their knowledge and skills (46.9% vs 18.4%, *P*=.009) and were 3.4 times more likely to worry about exams (68.1% vs 19.8%, *P*=.01; results not shown).

### Rural Location

Another finding was that there were differences in the results between students who were on placement in Dunedin and those who undertook placement outside of Dunedin. Some areas, such as the West Coast of the South Island, are the most isolated locations in New Zealand. Although anecdotal reports from students are that the learning and experiences in these isolated locations are extremely beneficial, the results from the study show that well-being scores are lower when students undertake a placement outside of Dunedin. Students undertaking Dunedin-based placements were 2.4 times more likely to receive constructive feedback compared with their colleagues based outside of Dunedin (*P*=.04). Students on placements away from Dunedin were 2.1 times as likely to experience stress or worry (*P*=.009; Table 2 OR results not shown) and in Multimedia Appendix 1, reported 0.6 lower (21%) scores in relation to the question about waking up feeling refreshed and well rested (Q4; *P*=.02).

### Well-Being Scores by Placement

**Figure 3:**
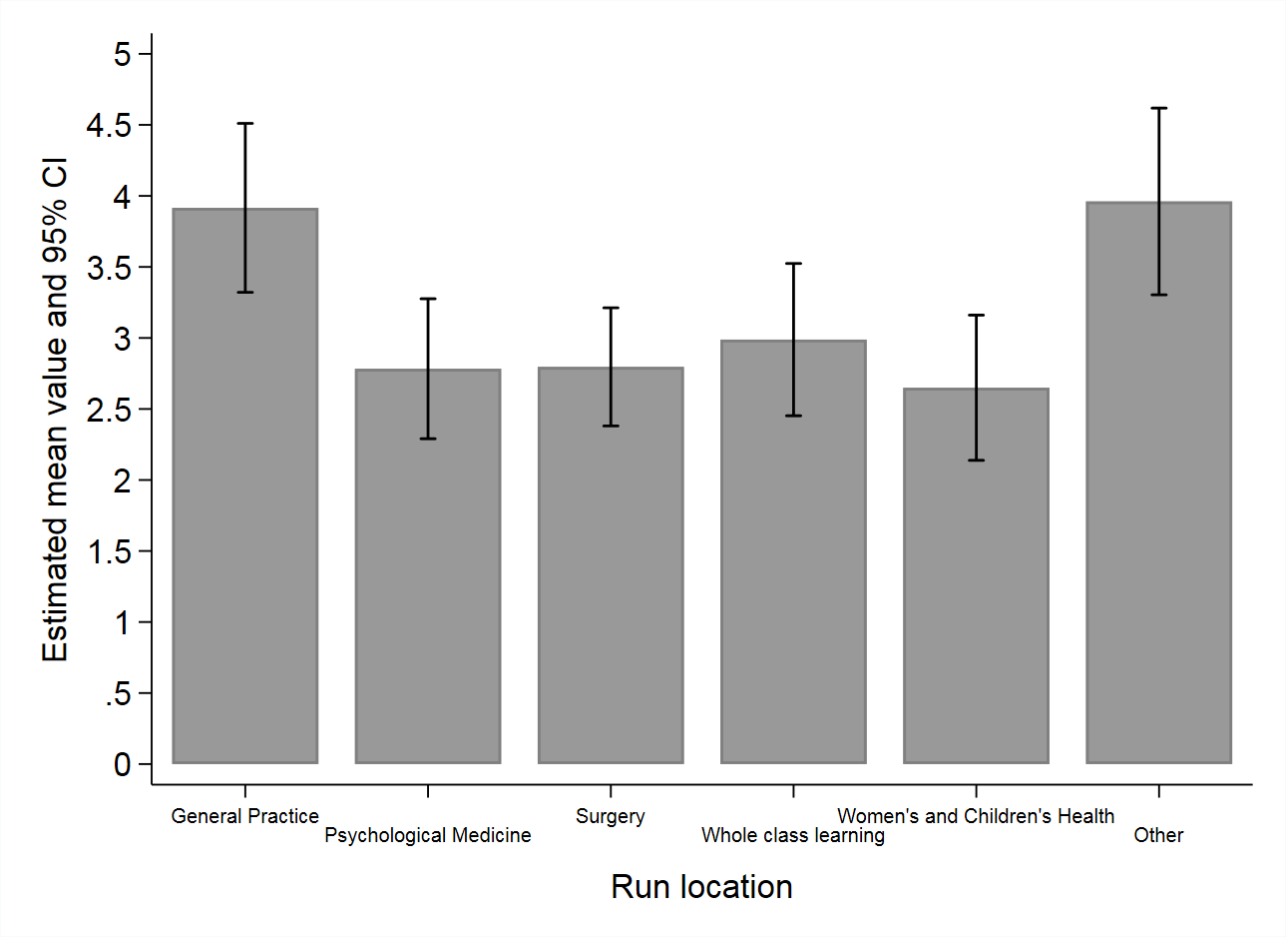
Mean well-being scores by placement.

There were apparent differences in well-being scores between students assigned to different specialties for their clinical placement (Multimedia Appendix 1, overall *P*<.001) (Figure 3). General practice scored highest of all the specialties (mean 4.02/5.00), followed by “other” (emergency department, intensive care unit, and public health; 3.78), and surgery (3.13). The lowest scores were reported by students undertaking the lecture-based whole class learning week (2.63), psychological medicine (2.63), and women and children’s health (2.51).

### Experiences Effect on Well-Being

Multimedia Appendix 2 details what participants experienced and the effect on well-being score. A total of five incidents of bullying or harassment were reported by students during the study. These incidents showed to have had a significant adverse effect (AE) on the participant’s well-being, and in particular, there was an AE recorded for three of the five questions; for example, feeling cheerful (Q1), feeling calm and relaxed (Q2), and day filled with things that interest me Q5). However, the daily score (*P*=.06; Multimedia Appendix 2) was not statistically significantly lower overall. “Receiving feedback that was not constructive or helpful” had the greatest impact on a participant’s overall well-being score, being associated with 1.18 lower mean scores, equivalent to a 37% reduction in a participant’s well-being (*P*<.001). Other large overall well-being decreases included “Felt like I didn’t learn anything” (29% lower for overall score, *P*<.001), followed by “Felt like I was treated unfairly” (21% lower for overall score, *P*<.001), and “stress or worry about something outside of medical training” (20% lower for overall score, *P*<.001). On the other hand, the recorded experiences that increased well-being scores were as follows: “Felt confident about my knowledge or skills” (19% higher, *P*<.001), “engaged in a hobby, sport, or social activity” (18% higher, *P*<.001), and “received feedback that was constructive or helpful” (17% higher, *P*<.001).

### Constructive Feedback

Due to the low levels of participants in the category “other specialities,” it is difficult to interpret these results. However, for the other placements, there was sufficient data to analyze. With respect to “receiving constructive feedback,” although there was no overall evidence for differences (*P*=.19), general practice was the highest at 40.0%, followed by surgery (30.5%), psych medicine (24.6%), and women and children’s health (24.4%). Not surprisingly, whole class learning week, which is lecture and small tutorial-based learning, had the least amount of constructive feedback for students (14.8%).

**Table 2:** Experiences versus mean well-being score.

Experience		Not experienced	1 or more days	Difference	*P* value
**Increased well-being**					
	Felt confident about my knowledge or skills	3.02	3.59	+0.57	<.001
	Engaged in a hobby, sport, or social activity	2.92	3.45	+0.53	<.001
	Received feedback that was constructive or helpful	3.05	3.57	+0.52	<.001
	Felt I learnt something new	2.96	3.41	+0.45	<.001
	Felt like I helped someone	3.05	3.45	+0.40	<.001
**Decreased well-being**					
	Received feedback that was not constructive or helpful	3.21	2.03	−1.18	<.001
	Felt I was bullied or harassed	3.41	2.33	−1.08	.005
	Felt I didn’t learn anything	3.28	2.34	−0.94	<.001
	Stress or worry about something outside of medical training	3.29	2.63	−0.67	<.001
	Felt I was treated unfairly	3.19	2.53	−0.66	<.02
	Worried about exams	3.37	2.74	−0.63	.001

### Qualitative Results

The results are from a narrative analysis of the focus group verbatim text and the “free-text” email surveys. Participants felt that using the Particip8 app to track well-being was feasible because they often had small amounts of time available to complete the survey.

#### Theme 1: Finding the Time

Some of the participants stated the following:

I feel like it would be quite easy because as a 4th year there is a lot of times there is waiting around and a lot of those times I tend to go on my phone, so I think that it will be easy to find time for a few minutes to go on [and do the Particip8app].

4th-year medical student participant

There is a lot of down time sitting outside out-patients, waiting for consultants, etc, it is a good way to use the time productively and easily.

6th-year medical student participant

Students are taught that regular reflection is important, but it is difficult to establish a habit. Participants also thought that an app would help establish these patterns and habits.

#### Theme 2: A Tool to Help Create Habits on Self-Reflection

Some of the participants stated the following:

I think that it would be difficult at the start to get into the habit of it, but, if I say do it every day at 5 o’clock, I would like to get into the habit of it, like doing it on the bus or something.

4th-year medical student, participant

...thinking about these questions every day, kind of makes you a bit more mindful of it, then you then, “ah,” like if you are having a particularly good day, “I've had a really good day,” or if things haven’t gone so well, you actually sort of think about that and sort of realise things that you might have otherwise missed.

4th-year medical student participant

#### Theme 3: Daily Reflection Increased Self-Awareness of Well-Being

Some of the participants stated the following:

Sometimes it is hard to know that you are actually quite stressed out.

4th-year medical student participant

I think as well that the environments that we’re working in the hospitals, can be quite stressful environments, so it’s important that we are able to take care of our well-being so that we are able to best respond to those stressful environments in a way that’s not going to be like self-destructive or damaging to ourselves.

4th-year medical student participant

I think that it is very important. It is the kind of the core of what we need to do, to do anything else you need to be well.

5th-year medical student participant

On the email poststudy survey questionnaire, students confirmed that their overall awareness of well-being had increased by 20% on a Likert scale from poststudy qualitative results.

### Analysis of the Safety Feature

Participants who had logged 3 days of low well-being scores triggered a safety feature on the Particip8 app, which alerted them and suggested places that were available for help and assistance. During the study, 41.7% (12/29) of all participants received the safety pop-up message at some stage during the study period. When asked if they sought support, most participants said that they talked to a trusted person, and 5 participants went to student health services. No participants stated that they had gone to the Medical School Associate Dean of Student Affairs for assistance.

After taking part in the study, over 90% (26/29) of students say a measure of their own wellness was useful. A further 75% (22/29) of participants said that they would be happy for their data to be reported back to the medical school faculty with some identification, such as demographics. The remainder of the participants (25%, 7/29) agreed to the data being reported to the medical school faculty on the provision that their data remained completely anonymous.

## Discussion

### Principal Findings

This study provides several important academic and practical outcomes. This feasibility study has examined the ability to collect self-reflected well-being data from medical student users via a smartphone app. The results collected, including focus group feedback and compliance percentages, show that this was overall a feasible method of collecting these data, although strategies to increase compliance would be advisable and worthwhile.

Prior studies in psychology suggest that use of the face emoticon scale can make participation more enjoyable. The study did not encounter any issues with regard to the inconvenience to participants to use the Particip8 app. A suggestion for the future development of the Particip8 app would be to add a dashboard page with a graph of a personal self-reflected well-being results over a week. Such a mechanism would allow participants to view the trends of their data and enable them to look back on past logged days themselves. The academic implication shown by this study is that surveys can be administered with ease and minimal burden to participants. This has potential generalized implications on future study methodologies, which require participants to complete short questionnaires at regular intervals.

The recorded well-being data was associated with experiences in ways that seem plausible, providing some degree of validation.

There are many challenges and practical implications that arise from conducting research by utilizing a smartphone app such as Particp8. Studies such as this are able to provide “real-time” data on the experiences of medical students and can generate a wealth of accurate prevalence data on well-being scores. However, the issue of “big data” and how to best analyze and interpret this becomes the next challenge.

### Limitations

Several limitations influence the conclusions and recommendations drawn from this research. First, the sample was small, self-selected, and drawn from a medical student population. This allowed, as intended, for a detailed exploration of an at-risk group who are likely to benefit from reflection of personal well-being. However, this adds limitations as the sample may differ from other young people, and the extent to which the themes discerned here are applicable across other populations or university groups is unclear. Second, there is also the limitation that comes with all self-reported data, whereby the participants may not be completely honest and candid in their reflections. One advantage of the methodology used in this study that counters this concern is that the daily survey detects change in the participants’ self-reflected well-being score when experiencing different situations. This comparison with the WHO-5 score increases the credibility of the self-reflections, rather than relying on the WHO-5 score alone.

Third, there may be concerns that the collection of data, with only a small number of survey questions, may not be adequate to accurately decipher trends. On the basis of findings from this research, it is argued that because daily reporting via the Particip8 app increases the amount of data received overall, this compensates for any disadvantage of the kind identified. Furthermore, the data collected from this study were sufficient to demonstrate several statistically significant results.

Finally, another concern is that daily self-reflection could become a burden or inconvenience to users of the Particip8 app. Users may become annoyed and resentful toward the Particip8 app’s daily “pop-up alerts” and push notifications to complete surveys. Despite this concern, the focus groups and poststudy results did not indicate any issue with annoyance or inconvenience. In fact, participants felt that because the survey could be usually completed in less than 1 min, the Particip8 app itself was not burdensome. Participants also noted that they appreciated the ability to complete the Particip8 app whenever they wanted, rather than at predetermined times stipulated by the researchers. Nonetheless, this feasibility study provides an initial understanding of the opportunities for successful smartphone-based collection of real-time self-reflected well-being data.

### Future Research

As this was only a feasibility study with a small sample size and was of a relatively short duration, future studies should investigate the feasibility over longer durations, in particular, to assess any further decline in compliance rates.

Future research is now focused on developing an updated Particip8 app. Such an app will have additional functions such as anonymous reporting of inappropriate behavior experienced. Notably, the anonymous reporting of inappropriate behavior is something that over half of participants said that they would find useful. Participants in the study also requested a “free-text” area so that they could write and record more detailed accounts of experiences during the day. This free-text area would be similar to a reflective journal and would be useful to the user.

Another potential area for further research is the incorporation of “interventions” into the Particip8 app. These could be either online interventions that are contained within the Particip8 app itself, such as a mindfulness recording, or could be “in-person” interventions, such as attending workshops. Any changes to a user’s baseline well-being could be monitored by real-time monitoring from the Particip8 app’s self-reflected well-being scores. Furthermore, the data from these studies could be used to determine whether there is any correlation between improvements in self-reflected well-being scores and reduced clinician burnout, depression, anxiety, and suicide. Ultimately, this would result in optimal patient outcomes in the long term.

Given the limitations of this study, its findings serve as research questions for future investigations and studies, rather than for providing definitive answers.

### Conclusions

In conclusion, the findings of this study suggest that the 28-day longitudinal collection of daily self-reflection well-being data via the Particip8 app was feasible. Further research is required to determine how to sustain the compliance with methodology over a longer period of time, as well as how to use the data to improve the well-being in clinical medical students.

## Appendix

Multimedia Appendix 1 Demographic and situational predictors of well-being scores.

Multimedia Appendix 2 Experiential predictors of well-being scores.

## Abbreviations

AE: adverse effect
EMA: ecological momentary assessment
HSFY: health science first year
LMM: linear mixed model
MBChB: bachelor of medicine and surgery
NZMSA: New Zealand Medical Students’ Association
